# Adverse Childhood Experiences and Life Course Adversity Related to Geriatric Syndromes: A Narrative Review

**DOI:** 10.3390/medicina62040618

**Published:** 2026-03-24

**Authors:** Carlos A. Reyes-Ortiz, Ximena Castro-Florez, Jose M. Ocampo-Chaparro

**Affiliations:** 1 The Institute of Public Health, College of Pharmacy and Pharmaceutical Sciences, Florida A & M University, Tallahassee, FL 32307, USA; 2 Geriatrics Specialty Program, Department of Family Medicine, Valle University, Cali 760042, Colombia; ximena.castro@correounivalle.edu.co (X.C.-F.); jose.m.ocampo@correounivalle.edu.co (J.M.O.-C.)

**Keywords:** geriatric syndromes, older adults, adverse childhood experiences, abuse, maltreatment, discrimination, stressful events, life course

## Abstract

Background/Objectives: During the last decades, there has been a growing interest in and knowledge of the relationship between adverse childhood experiences (ACEs) or stressful life events and health issues in younger and adult populations. However, less is known in older populations. This narrative review aimed to summarize cross-syndrome studies on the relationship between life course exposure to adversity (e.g., ACEs, discrimination, famine, adult violence) and geriatric syndromes (e.g., dementia, frailty, falls, depression), with the primary intention of providing a descriptive mapping of the evidence and identifying gaps. Methods: We searched PubMed for articles published between 2015 and 2026 using specific keywords. Results: We included 84 studies. There is substantial variability in the exposure measures used (e.g., for ACEs, from one question to more than 30) and in the outcomes (e.g., different diagnostic criteria for dementia or frailty). Conclusions: Our synthesis showed that ACEs, stressful events, and other adversity measures are usually associated with greater probabilities of the occurrence of geriatric syndromes such as dementia, frailty, depression, falls, low muscle strength, multimorbidity, and functional decline. There are also some reports for mediators, depression being the most common, that may partially explain those associations.

## 1. Introduction

Geriatric syndromes are a group of diverse health conditions commonly observed in older adults, less frequently in younger adults, and not classified as specific diseases [1,2]. Geriatric syndromes in older people are also considered common, multifactorial health conditions resulting from accumulated impairments across the life course in multiple systems [3,4]. Bernard Isaacs (cited by Morley) [5] was the first to mention the major geriatric syndromes, or ‘Giants of Geriatrics’, as instability (replaced later as falls), immobility (later equivalent to functional dependence), incontinence (urinary or fecal), and intellectual impairment (named later as cognitive impairment, including dementia and delirium) [1,2,3,4]. Then, geriatricians and researchers included other geriatric syndromes such as frailty, sarcopenia, dizziness, syncope, functional decline, low strength, pressure ulcers, depression, polypharmacy, and multimorbidity [6,7,8,9].

Our central hypothesis for this narrative review is that multilevel life course exposures (e.g., at the individual, family, and various environmental levels) during the prenatal period, childhood, youth, early adulthood, late adulthood, or old age may be associated with the long-term development of geriatric syndromes in older adults. Our hypothesis is based, in part, on combining the life course approach [10,11] and the social–ecological model [12], both of which are very useful in public health and aging [13,14,15]. The life course exposures include traumatic or stressful life events, adverse childhood experiences (ACEs) (e.g., abuse, household dysfunction), deprivation (e.g., parental neglect), or adversity in social determinants of health (e.g., poverty, poor health status, hunger) [16,17]. Many studies have focused on youth or adulthood [18,19,20], but fewer have examined the consequences of lifetime exposures for middle-aged or older people [21,22]. Potential risk exposures include traumatic events (e.g., early parental death), chronic stress (e.g., domestic violence), neighborhood environment (e.g., unsafe), socioeconomic adversity, isolation, bullying, and discrimination [16,17,18,19,20,21,22].

This narrative review aimed to summarize studies on the relationship between life course exposures and geriatric syndromes. Existing work typically focuses either on single outcomes (e.g., frailty or dementia) [21,22] or on single exposure constructs (e.g., ACEs) [16,18]. By contrast, this manuscript attempts to provide context, cross-syndrome synthesis, and cross-level integration. Our narrative review also aimed to address its implications for public health and geriatric medicine, including the importance of early-life prevention and trauma-informed geriatric care.

## 2. Materials and Methods

We performed the search in PubMed/Medline and limited it to papers reporting in English. Since the scope of our narrative review was to report contemporary trends, we searched papers published between 1 January 2015 and 31 January 2026. We chose PubMed/Medline, a simple, publicly available [no registration, etc.] database with an easy-to-use citation format (e.g., APA, AMA).

The principal words we searched are as follows: “(Geriatric Syndrome) OR (Cognitive decline) OR (Cognitive impairment) OR (Dementia) OR (Frailty) OR (Falls) OR (Sarcopenia) OR (Muscle strength) OR (Functional decline) OR (Functional limitations) OR (Delirium) OR (Depression) OR (Multimorbidity)” AND “(Adverse Childhood Experiences) OR (Childhood Adversity) OR (Early Adversity) OR (Early Life Stress) OR (Violence) OR (Stressful Events) OR (Maltreatment) OR (Abuse) OR (Discrimination) OR (Traumatic Events) OR (Neighborhood Deprivation)”. Search strings were not pilot tested.

**Figure 1:**
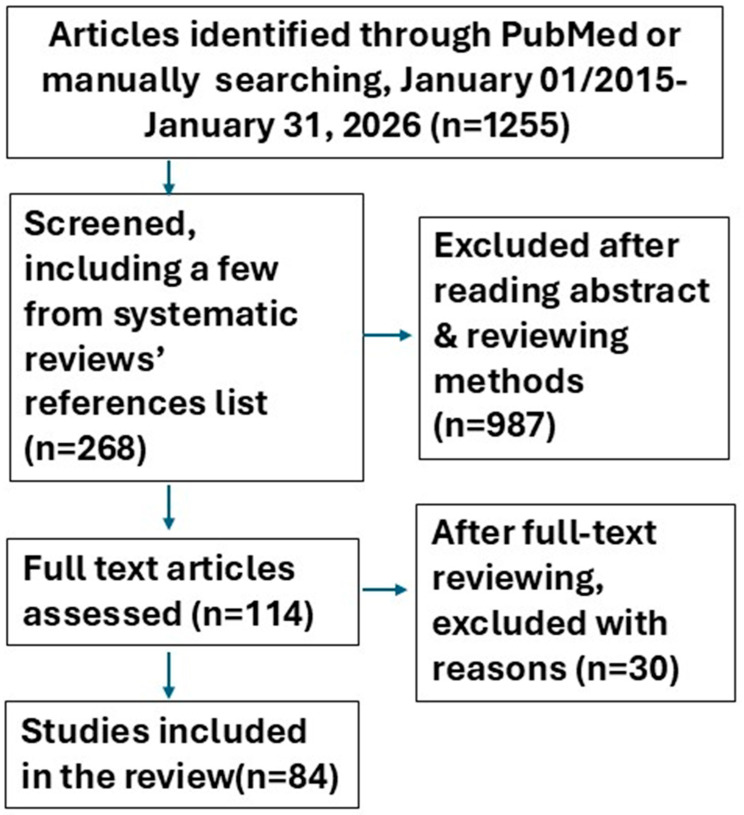
Flowchart of included studies.

Inclusion criteria were (1) studies that included original research and (2) articles reporting at least one outcome of interest (a geriatric syndrome) with specific exposures related to ACEs, stressful or traumatic life events, adversity, abuse, deprivation, discrimination, or similar during the life span (the whole period or parts from the prenatal stage to old age). We also searched systematic reviews and the reference lists of the initially included studies and manually reviewed them to identify other potentially relevant publications. Only published, anonymized data were used. However, given the sensitive nature of ACEs and violence data, readers need to exercise caution when interpreting or applying our findings. We excluded non-original studies, abstract-only publications, duplicates, articles including adults but not older adults, and articles in languages other than English. We also excluded studies that only assessed adverse events in adulthood or health outcomes in children. The flowchart of our search is in Figure 1.

## 3. Results

We included 84 studies and summarized them in eight tables. Each table includes columns with 1 First author’s last name with year of publication and country; 2 Study design and population; 3 Exposures; 4 Geriatric syndromes or other outcomes; 5 Main outcome’s findings; and 6 Additional comments. Studies are listed by year of publication, from the oldest to the newest.

### 3.1. Dementia or Cognitive Impairment

Twenty-one studies on dementia or cognitive impairment published from 2018 to 2025 are listed in Table 1 [23,24,25,26,27,28,29,30,31,32,33,34,35,36,37,38,39,40,41,42,43]. Early life stress was associated with dementia and poorer global cognition [23,26]. Early parental (mother or father or both) deaths were associated with increased odds for dementia [24] and lower cognitive function [27,30]. Poor childhood health was associated with lower cognitive function [27]. Childhood maltreatment was associated with dementia [29]. Documented abuse and neglect were associated with cognitive impairment [39]. A greater number of ACEs was associated with worse cognition [30,31,38], dementia [25,32,37,43], and subjective cognitive decline [34]. Psychosocial stressors (e.g., financial strain, neighborhood disadvantage, poor socioeconomic status [SES]) were associated with dementia and cognitive impairment [40,41,42]. Women with lifetime post-traumatic stress disorder (PTSD) and re-experiencing symptoms for PTSD had higher odds for low global cognition. However, men had lower odds for low global cognition [28]. Life course exposure to traumatic events was associated with cognitive decline [33,36] and dementia [35]. As a methodological summary, in this Table, only one study is case-control [24], and another is cross-sectional [34]. The rest are cohorts. One study included lifetime or current PTSD [28]. Two studies included lifetime [33,36,40] or recent (at older age) [35,41] traumatic events (e.g., disasters, adult violence, socioeconomic deprivation, illness) as exposures. One study included documented cases of abuse/neglect as exposure [39]. All other ACEs or early life stress (e.g., abuse/neglect or socioeconomic deprivation) exposures are retrospective. Nine studies used dementia diagnosis as the geriatric syndrome outcome [23,24,25,28,29,32,37,40,43], but each used a different diagnostic criterion. For example, a doctor’s diagnosis (or health records/ICD-10 diagnosis) [23,24,37], MMSE score [25], DSM-IV criteria [28,32,35], TICS-m [33,43], AD-8 [29], and Langa–Weir classification [40]. Other studies have cognitive impairment as an outcome based on the MMSE or a series of other neuropsychological tests.

### 3.2. Frailty

Fifteen studies on frailty published from 2018 to 2025 are in Table 2 [44,45,46,47,48,49,50,51,52,53,54,55,56,57,58]. In two Finnish studies, exposure to early life stress, represented by wartime parental separation, was associated with increased frailty (measured by Fried’s criteria phenotype) among older men in a case-control study [49] and among women from midlife into old age in a longitudinal study [51]. Exposure to childhood starvation, food deprivation, or hunger [47,48,53] was associated with frailty (measured by the Frailty Index or the FRAIL Scale) among Chinese older adults. Lifetime exposure (fetal or school-age) to famine was associated with frailty (Frailty Index) in Chinese older adults [58]. Childhood adversity (abuse or neglect) was associated with frailty (Frailty Index) among adults aged 40–69 in the United Kingdom, and unhealthy lifestyle was a mediator [57]. Life course physical abuse (childhood) and psychological violence (by their intimate partner) were associated with frailty in older adults in a multinational study [45]. Lifetime neighborhood social deprivation was associated with frailty (Frailty Index) in older adults residing in Scotland, United Kingdom [49]. By contrast, better childhood neighborhood quality and better childhood health status were associated with lower odds of frailty in older age [47]. In three longitudinal studies, ACEs were associated with frailty (Frailty Index) among middle-aged and older adults [54,55,56]. In a Canadian cross-sectional study, ACEs were associated with frailty (Frailty Index) among older adults [52]. In a European longitudinal study, ACEs and adverse childhood health experiences were associated with frailty (Fried’s phenotype) among middle-aged and older adults [46]. In a Chinese longitudinal study, ACEs were associated with greater frailty [50]. In comparison, better self-rated childhood health and health care were associated with less frailty among middle-aged and older adults [50]. In summary, in this Table, only one study is a case-control [44], five are cross-sectional [45,52,53,57,58], the rest (nine) are cohorts. Seven studies reported early life exposure to war/famine [44,46,47,48,51,53,55], and one study reported lifetime (fetal, preschool, and school-aged) exposure to famine [58]. One study examined lifetime (childhood, young adulthood, late adulthood) exposure to socioeconomic deprivation (neighborhood social deprivation) [49]. Two other studies had socioeconomic deprivation and neighborhood quality exposures [54,56]. One study had lifetime (childhood, adulthood) violence exposure [45]. All ACEs or early life stress (e.g., abuse/neglect, household dysfunction or socioeconomic deprivation) exposures are retrospective. Among studies that included ACEs as exposures, three studies had only one ACE [44,51,53] such as wartime parental separation or childhood hunger. Four studies had Frailty based on Fried’s phenotype as the outcome [44,45,46,47]. One study had the FRAIL scale as the outcome [48]. One study had the LASA-Frailty Index scale [containing 32 deficits] as the outcome [55]. Nine other studies had the Frailty Index scale as the outcomes [49,50,51,52,53,54,56,57,58]; in these studies, the scale ranged from 30 to 76 deficits.

### 3.3. Depression

Fourteen studies on depression published from 2015 to 2026 are listed in Table 3 [59,60,61,62,63,64,65,66,67,68,69,70,71,72]. In two studies, childhood forced sexual intercourse [59] or ever being a victim of sexual abuse (along with being displaced by violence) [61] were associated with later life depression. In two studies, having poor or moderate perceived social support [60] and poor childhood friendship experiences [71] were associated with high depressive symptoms among older adults. In one study, childhood traumatic events and their severe impact on the current lives of Chinese older adults were associated with depressive symptoms score, and resilience mediated that relationship [65]. In older adults from England [62], Japan [63], China [64,66,68,70,72], the United States [67], and Korea [69] a higher number of ACEs was associated with depressive symptoms. In one English longitudinal study, greater ACEs exposure was associated with depressive symptoms, and CRP (inflammation marker) levels were a mediator [62]. In a Chinese longitudinal study, in addition to ACEs, adverse adult experiences (AAEs) like prolonged bed rest or lifetime discrimination were associated with depressive symptoms [68]. In some studies, other mediators between ACEs and depression were adult SES [63], arthritis [66,68], digestive or respiratory diseases [66], sleep duration [64,70], and other chronic diseases [70]. In summary, eight studies are cross-sectional [59,60,61,64,65,67,69,70], and six are cohorts [62,63,66,68,71,72]. Two studies have only one exposure: the first the abuse/neglect domain (ever being a victim of sexual abuse) [61], the second the social network domain (poor childhood friendship) [71]. One study assessed childhood traumatic events and their current impact (severity) in the participants’ lives [65]. All other ACEs or early life stress (e.g., abuse/neglect, household dysfunction, loss experiences, or socioeconomic deprivation) exposures are retrospective. One study used a self-reported history of depression as the outcome [67]. Two studies used the Patient Health Questionnaire (PHQ-8, PHQ-9) [59,69] as the depression measure outcome. Three studies used the Geriatric Depression Scale (GDS-15) as a measure of depressive symptoms [61,63,65]. Most Chinese studies (CHARLS) used the CES-D-10 scale to measure depressive symptoms [64,66,68,70,71,72]. Two other studies used different versions of the CES-D scale [60,62].

### 3.4. Falls

Seven studies on falls from 2018 to 2025 are in Table 4 [73,74,75,76,77,78,79]. Older adults with several abuse types (ever being abused: emotional, physical, sexual) and polyvictimization had increased odds for recurrent falling [73,74]. Older adults who had experienced lifetime discrimination (ever had, during childhood, or recently [within the last 5 years]) [75] or ageism (perceived age discrimination) [76] had increased odds of recurrent falling. Middle-aged and older adults exposed to more ACEs had higher odds of falling [77,78,79]. The most common mediator was depression [74,76,77,78,79], followed by functional decline and multimorbidity [76,79]. In summary, six studies are cross-sectional [73,74,75,76,78,79], and only one is a cohort [77]. Two studies assessed discrimination as an exposure: one by lifetime discrimination (childhood, ever, or recent) [75], and another by older age period [76]. Polyvictimization (≥2 types of abuse/neglect) [73,74,79] and poly-dysfunction (≥2 types of household dysfunction) [79] were also assessed exposures. In two Chinese studies, ACEs exposures included abuse/neglect, loss, an unsafe neighborhood, and adversity domains [77,78]. Falls during the past 12 months [73,74,75,76,79] were the most common outcome, followed by falls since the last survey (within about 2 years) [77,78].

### 3.5. Low Muscle Strength or Sarcopenia

Six studies on low muscle strength or sarcopenia published from 2018 to 2025 are in Table 5 [80,81,82,83,84,85]. In one longitudinal study, the less disadvantaged in early life had lower odds of lower handgrip strength than the most disadvantaged [80]. In another study, men with childhood adversities showed a steeper decline in handgrip strength over time [82]. In a cohort study, greater ACE exposure was associated with increased odds of low muscle strength among women but not among men [81]. In a cross-sectional study, a higher number of ACEs was associated with increased odds of low muscle strength [83]. In a longitudinal study, a higher number of ACEs (all or those with either threat or deprivation related) was associated with increased hazard ratios of sarcopenia [84]. By contrast, in a cross-sectional study, ACEs showed a protective association with sarcopenia among the oldest participants (75–85 years) [85]. In summary, this Table includes two cross-sectional studies [83,85], and four cohort studies [80,81,82,84]. ACEs exposures were all retrospective and included domains such as abuse/neglect, loss, neighborhood safety, adversity, and socioeconomic deprivation. In four studies, handgrip strength was the outcome [80,81,82,83]. In two studies, sarcopenia (diagnosed according to international guidelines) was the outcome [84,85].

### 3.6. Multimorbidity

Five studies on multimorbidity published from 2019 to 2024 are in Table 6 [86,87,88,89,90]. Any ACEs were associated with somatic and psychiatric multimorbidity among middle-aged adults but not among older adults in a US study [86]. Middle-aged and older adults exposed to a higher number of ACEs had higher odds of multimorbidity [87] and an increased number of chronic diseases [90] in two Chinese studies. A lifetime exposure to racial discrimination was associated with higher odds for multimorbidity among Colombian older adults [88]. ACEs related to childhood health were associated with multimorbidity from middle age onward in a Scottish longitudinal study [89]. In summary, three studies are cross-sectional [86,87,88], and two are cohorts [89,90]. ACEs exposures were all retrospective and included domains such as abuse/neglect, loss, neighborhood safety, adversity, and socioeconomic deprivation. One study, in addition to ACEs, included past year stressful life events as exposures [86]. One study assessed discrimination as a lifetime exposure (childhood, ever, or recent) [88]. Multimorbidity as the outcome was used as a binary (≥2 chronic conditions vs. 0–1) [87,88] or as a continuous variable [86,89,90].

### 3.7. Functional Decline

Four studies on functional decline from 2016 to 2023 are listed in Table 7 [91,92,93,94]. Middle-aged and older adults exposed to childhood physical abuse or violence had higher odds for poor lower extremity functioning [91,94]. Middle-aged and older adults exposed to a higher number of ACEs had increased odds of limitations of instrumental activities of daily living (IADL) [92] and activities of daily living (ADL) [93]. Chronic conditions and depression were mediators as potential pathways in two studies: first in the relationship between physical abuse and lower extremity functioning [91] and second in the relationship between ACEs and ADL scores [93]. One study included lifetime exposures in childhood (physical abuse) and adulthood (intimate partner/family member violence) [91]. In summary, three studies are cross-sectional [91,92,94] and one is a cohort [93]. ACEs exposures were all retrospective and included domains such as abuse/neglect, loss, illness, neighborhood safety, adversity, and socioeconomic deprivation [91,92,93,94]. One study, in addition to ACEs, included adulthood experiences of violence as exposures [91]. Lower extremity functioning was the outcome in two studies [91,94]. IADL and ADL limitations were outcomes in three studies [92,93]. Chronic conditions [91,93], depression [92], and adult health behaviors/health status [92] were mediators.

### 3.8. Other Diagnoses or Several Geriatric Syndromes

Twelve studies on other diagnoses or several geriatric syndromes from 2019 to 2026 are in Table 8 [95,96,97,98,99,100,101,102,103,104,105,106]. In an American study, child maltreatment was associated with adult interpersonal violence and later with elder abuse [95]. In a Bhutanese study, ACEs (WHO version) were associated with lung disease, vision impairment, insomnia, high blood pressure, and memory decline [98]. In another American study, ACEs were associated with lower sleep quality [99]. In a Chinese longitudinal study, a higher number of ACEs was associated with pain, chronic diseases, falls, multimorbidity, ADL, and IADL limitations [100]. In four Chinese cross-sectional studies, greater ACE exposure was associated with chronic respiratory diseases (chronic bronchitis, emphysema, and asthma) [101,103], anemia [102], and musculoskeletal pain [104]. In a Chinese study, fetal exposure to severe famine was associated with higher odds for chronic kidney disease [97]. In a South African study, life course exposure to traumatic events (e.g., community violence) was associated with depressive symptoms, PTSD symptoms, and ADL limitations [96]. In one Chinese longitudinal study, ACEs were associated with dementia and AAEs were associated with stroke at follow-up; in these associations, depressive symptoms were mediators [105]. In a US clinical study, ACEs were associated with severe obstructive defecation syndrome [106]. In summary, three studies are cross-sectional [95,96,97,98,99,101,102,103,104,106], and two are cohorts [100,105]. ACEs exposures were retrospective and included domains such as famine/fetal, abuse/neglect, loss, illness, neighborhood safety, adversity, socioeconomic deprivation, and adult violence [97,98,99,100,101,102,103,104,105,106]. One study included lifetime exposures such as child maltreatment and adulthood violence [95]. One study included lifetime exposure to famine (fetal, pre-school, school-aged) [98]. One study included lifetime traumatic experiences (e.g., childhood environment, community violence, illness/accident/disaster, social/family environment, and war violence) [96]. The outcomes included elder abuse, sleep quality, depression, dementia, stroke, functional decline, falls, pain, lung diseases, chronic kidney disease, chronic respiratory disease, anemia, obstructive defecation syndrome, and other chronic diseases. Depression was a mediator in four studies [100,101,104,105].

## 4. Discussion

This narrative review summarizes studies on the relationship between life course exposures and geriatric syndromes. Our review showed that ACEs and other adversity measures across life are associated with greater probabilities for the occurrence of geriatric syndromes such as dementia, frailty, depression, falls, low muscle strength, multimorbidity, and functional decline.

**Figure 2:**
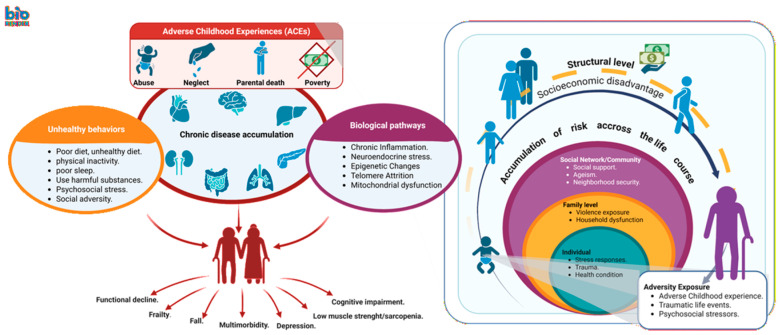
Conceptual pathways linking life-course adversity to common geriatric syndromes. Created with Biorender.com.

Using the life course approach and the eco-social model together suggests that biological, psychological, behavioral, social factors, and environments act throughout the aging process and the older person’s life, are biologically embedded and modify health and functional status over time and are associated with many geriatric syndromes [10,11,12,13,14,15]. Figure 2 depicts our conceptual framework synthesis based on the life course perspective periods (e.g., perinatal through later life) and eco-social perspective levels (e.g., individual, family, social network, neighborhood) applied to the relationship between life cycle adversity exposures (e.g., ACEs, abuse, discrimination, violence, traumatic events, deprivation) and resulting geriatric syndromes (e.g., dementia, frailty, depression, falls, and others) [10,11,12,13,14,15,16,17,18,19].

ACEs and other forms of cumulative adversity may act through multiple interconnected mechanisms. These include behavioral pathways (e.g., unhealthy lifestyle patterns such as poor diet, physical inactivity, sleep disturbances, and substance use), chronic disease accumulation, and biological processes associated with stress and aging. Biological pathways described in the literature include chronic inflammation, neuroendocrine dysregulation related to stress responses (at the hypothalamic-pituitary-adrenal (HPA) axis with triggering cortisol release), allostatic load, epigenetic modifications, telomere attrition, and mitochondrial dysfunction [16,17,18,19,107,108,109,110,111,112]. Together, these lifetime and multilevel pathways may increase vulnerability to the listed geriatric syndromes.

In Table 9, as an additional conceptual synthesis, based on studies presented in Table 1, Table 2, Table 3, Table 4, Table 5, Table 6, Table 7 and Table 8, we include examples from our results to integrate life course stages and life course epidemiology frameworks (e.g., risk accumulation, critical/sensitive periods, pathway models) [10,11,15] with the standardized exposure domains (e.g., famine, abuse/neglect, adversity, household dysfunction, socioeconomic deprivation) related to geriatric syndromes, and we mention some diagnoses or syndromes found in this narrative review as mediators.

Thus, our results in this narrative review support the idea that many geriatric syndromes are associated with early life exposures from the perinatal period through later ages. Therefore, health professionals should consider early exposures at several levels, such as individual (e.g., victim of abuse), family (e.g., violence or deprivation), social network (e.g., peer bullying), and neighborhood environments (e.g., unsafety) in the pathways of common geriatric syndromes throughout the entire life course from the perinatal period (e.g., famine), followed by childhood and adolescence (e.g., parental deaths), adulthood (e.g., unemployment), and later life (e.g., illness) [10,11,12,13,14,15,79]. Moreover, it is essential to understand the full impact of early adversity on geriatric syndromes later in life and to design interventions that address the multiple levels of influence to break cycles of harm.

In public health and geriatric medicine, preventing ACEs and discrimination in early life periods will be ideal because they interact in a syndemic way with other lifetime adversities and social inequalities, increasing the possibilities of becoming ill or dying [113,114]. For example, during the perinatal and early childhood periods and across both individual- and family-level factors, the negative effects of ACEs or discrimination can be mitigated through parenting-skills training, preschool environment enrichment, and early childhood home visitation programs [13,115,116,117]. Efforts to reduce socioeconomic inequalities by creating healthy environments and promoting children’s development can also mitigate the negative effects of ACEs [13,19,115,116,117]. In addition, during adolescence, adulthood, and later life periods, cultivating positive peer relationships, social networks, and neighborhood support is essential for maintaining adequate social participation, physical activity, and functionality, along with raising awareness of the risk factors for geriatric syndromes (e.g., risk factors for falls such as vision problems, depression, multimorbidity, memory difficulties, and functional decline usually start at middle age and continue through older ages) [79]. In addition, public health and gerontology efforts are needed to create policies related to programs for trauma exposure prevention and management, along with safe environments by increasing prevention and management of family (e.g., abuse/neglect), school (e.g., bullying), and community violence (e.g., unsafe neighborhoods) [13,19,115,116,117].

For assessment, one initial strategy could be to ask older adults about positive experiences during early life and later, followed by the expression of potential negative events [19]. Although ACEs and discrimination assessments are considered sensitive issues [19], they could be incorporated in the comprehensive geriatric assessment, in the psychosocial or mental component, like the elder abuse tool assessments that have been used and validated in population-based studies, such as “Have you ever been a victim of… abuse?” [61,73,74] or “Have you felt rejected or discriminated…?” [75,76,88]. These questions should be asked to the older person in a separate room if he/she lives with someone. However, when detecting exposure to ACEs or discrimination, it is always important to have an interdisciplinary approach, involving mental health professionals or geriatricians trained in abuse/neglect assessments [118], if requiring deep evaluation and management.

Some other models may also help. The SAMSHA Trauma-Informed Approach includes six key principles such as 1–Safety (e.g., keeping privacy, feeling support); 2–Trustworthiness and transparency (e.g., building rapport and trust); 3–Peer support (e.g., providing information in person or online support from trauma survivors); 4–Collaboration and mutuality (e.g., partnering with other staff, caregivers or family members); 5–Empowerment, voice, and choice (e.g., assuring the patient of the option to end the assessment at any point); and 6–Cultural, historical, and gender issues (e.g., guided by principles of cultural humility) [19,119,120].

Therefore, for early detection of ACEs or discrimination exposure, geriatricians and other clinicians should routinely ask about or screen for ACEs, especially those exposed to racism, polyvictimization, or poly-dysfunction, and when an ACE is detected, provide or refer for incorporating trauma-informed care for those affected [19,117,120]. On the other hand, given the complexity of depression diagnosis and clinical presentation in older adults (e.g., mimicking of somatic conditions without sadness) and because it is linked to many geriatric syndromes and a common mediator (as we describe in this review) the relationship between ACEs and discrimination and several geriatric syndromes [37,42,74,76,77,78,79,91,100,101,104,105] make depression an important target for prevention, early detection, and treatment across the lifetime periods.

As strengths, this narrative review provides a broad overview of studies examining the relationship between multiple life course exposures, from the prenatal period to older age and geriatric syndromes. These life course exposures occur across individuals, families, and neighborhoods. We also identified mediators [the most common was depression] as potential modifiable pathways between the exposures and the geriatric syndromes.

However, this narrative review has some limitations. We did not use a systematic approach to article selection because of the heterogeneity in study designs, exposures, and reported outcomes; consequently, these results are not suitable for causal interpretation. Articles have been incorporated based on the authors’ assessment of relevance, potentially introducing selection bias. In addition, the heterogeneity of the included studies (especially in terms of exposures and outcomes), along with differences in design, population, and methodological procedures, makes any direct or statistical comparison impractical. Future reviews may focus on selected geriatric syndromes as outcomes (e.g., with equivalent diagnostic criteria), on similar exposures (e.g., only ACEs), and on cohort studies.

Other potential methodological limitations of our narrative review include restricting ourselves to a single database search, excluding non-English literature, recall bias (e.g., most ACEs are retrospective measurements, although responses are generally stable over time [121]), survival bias in older populations [e.g., ACEs had a protective effect on sarcopenia among the oldest group (75–85 years) in a Canadian study, likely related to survival of the fittest [85] (or there could be measurement issues or contextual (cohort, cultural) differences), as an example of conflicting results that we wanted to report in the Tables, since sometimes the associations may go in unexpected directions], publication bias (highlighting positive results), reverse causality (in cross-sectional analyses) and overrepresentation of certain cohorts (e.g., CHARLS, HRS), or residual confounding (unmeasured confounding factors, due to lack of randomization, a threat to the validity of observational studies). Future systematic reviews should conduct formal risk-of-bias or quality appraisals and use a standardized framework to grade the strength or certainty of the evidence. However, due to the nature of the exposures (ACEs, abuse, discrimination), which are not amenable to experimental or quasi-experimental research because they are considered unethical, we will need more consistent observational studies (e.g., longitudinal) to build further evidence on these topics.

## 5. Conclusions

In conclusion, our synthesis showed that ACEs and other adversity measures during the life course are usually associated with greater probabilities of the occurrence of geriatric syndromes such as dementia, frailty, falls, sarcopenia, functional decline, depression, and multimorbidity. There are also some reports for mediators that may partially explain those associations. In some areas (e.g., ACEs and depression, ACEs and frailty, ACEs and dementia) the evidence is relatively consistent and robust, but in other areas the findings are more mixed or sparse (e.g., sarcopenia, complex multimorbidity patterns). Finally, key future research priorities include standardized adversity measures, harmonized frailty definitions, and longitudinal analyses starting earlier in life.

## Figures and Tables

**Table 1:** Studies related to Dementia or Cognitive Impairment.

Authors	Design, Population	Exposures	Geriatric Syndromes or Other Outcomes	Main Outcome’s Findings	Additional Comments
Donley, 2018 [23], Finland	Longitudinal cohort, 2682 men aged ≥42; KIHD	Childhood stress (interviews 1984 and 1989) included living in custody or an orphanage, experience of crisis in childhood, having problems with teachers, and emigrating because of war	Incident cases of dementia, including AD, were obtained through 2014 via national health register linkages	Childhood stress was associated with increased risk of dementia (HR = 1.93 95% CI 1.14–3.25).	
Conde-Sala, 2020 [24], Spain	Case-control study 65, 997 participants aged ≥50; SHARE	Parental death (mother or father or both) at the age of ≤16 years (childhood and adolescence)	Diagnosis of dementia by a doctor	Early parental death associated with increased odds of dementia (OR = 1.50 95% CI 1.31–1.72).	
Tani, 2020 [25], Japan	Longitudinal (3-yr follow-up), 17,412 participants aged ≥65; JAGES	ACEs 7 questions, before the age of 18 interpersonal loss (parental loss or parental divorce), family psychopathology (parental mental illness or family violence), and abuse and neglect (physical abuse, psychological neglect, or psychological abuse).	Dementia was defined as level II or higher on the dementia scale (it corresponds to a 16-point rating on the Mini-Mental State Examination)	Participants who experienced ≥3 ACEs (vs. 0) had a greater risk of developing dementia (HR = 1.78 95% CI 1.15–2.75; *p* = 0.009)	
Grainger, 2020 [26], Australia	Cohort study, 484 older adults (mean age 83.4 SD = 4.3); Sydney MAS	Early life stress (ELS), using the short form of the Childhood Trauma Questionnaire, includes 28 items and assesses 5 traumatic experiences: physical abuse, physical neglect, sexual abuse, emotional abuse, emotional neglect.	Global cognitive function measured by 10 neuropsychological tests with domains: attention/processing speed, memory, language, visuospatial reasoning, and executive functioning. (standardized z-scores)	Global cognition was poorer in those who had experienced physical neglect (mean −0.48 SD = 1.04) relative to those who had not (mean −0.14 SD = 1.08; *p* < 0.05).	
Yang, 2020 [27], China	Longitudinal, 16,258 participants aged ≥45; CHARLS	Early life conditions (ELCs): early parental death, childhood SES, food deprivation, and childhood health	Cognitive function was assessed using episodic memory (immediate and delayed recall) and mental intactness (serial subtractions, date, day of week, redraw a picture)	Early maternal death was associated with lower cognitive function among middle- and old-aged Chinese adults (β range between −0.44 and −0.35, *p* < 0.05). Less healthy participants during childhood had lower cognitive performance than those who had enjoyed good health (β range between −0.36 and −0.14, *p* < 0.05).	
Nilaweera, 2020 [28], France	Longitudinal (14 years), 1700 participants aged ≥65, ESPRIT study cohort	Lifetime and current post-traumatic stress disorder (PTSD) diagnoses were assessed using the Watson’s PTSD Inventory	Cognitive tests assessed global cognition, visual memory, verbal fluency, psychomotor speed, and executive function. Incident dementia (Dx DSM-IV criteria)	Men with lifetime PTSD or without re-experiencing symptoms had lower odds for low global cognition (OR = 0.61 95% CI 0.42–0.88; and OR = 0.57 95% CI 0.38–0.84, respectively). Women with re-experiencing symptoms had higher odds for low global cognition (OR = 1.46 95% CI 1.03–2.09)	Women without re-experiencing symptoms had lower risk for dementia (HR = 0.49 95% CI 0.29–0.80)
Macpherson, 2021 [29], UK	Retrospective cohort study 56,082 people age 55.5 (±7.7); UK Biobank	Childhood maltreatment (CM) included physical abuse, physical neglect, emotional abuse, emotional neglect, and sexual abuse	Incident dementia diagnosis—AD-8: The Washington University Dementia Screening Test	CM was associated with dementia (HR = 1.32, 95% CI 1.02–1.71)	
Gold, 2021 [30], USA	Cohort 1661 participants aged ≥65; KHANDLE	ACEs (score 0–9) before age 16: parents divorced; parents remarried. Domestic violence; family member substance abuse or serious illness, parent loss of a job; parent was in jail, death of mother or father.	Spanish and English Neuropsychological Assessment Scales (SENAS) including Verbal episodic memory, semantic memory, and executive function	ACEs associated with worse cognition: parent remarried (β = −0.11 95% CI −0.20 to −0.03); death of mother (β = −0.18 95% CI −0.30 to −0.07); and death of father (β = −0.11 95% CI −0.20 to −0.01)	ACEs total score was not associated with cognition
Lin, 2022 [31], China	Prospective cohort, 6466 participants mean age 57.2 (SD = 8.3); CHARLS	ACEs 10 types, 5 threat-related adversities (i.e., physical abuse, household substance abuse, domestic violence, unsafe neighborhood, bullying) and 5 deprivation-related adversities (i.e., emotional neglect, household mental illness or someone incarcerated, parental divorce, or death)	Episodic memory (immediate and delayed recall (score 0–10). Executive function (orientation, calculation and visuospatial ability (score 0–11). Global cognition (total score 0–21)	Participants with ≥2 childhood deprivations had faster cognitive decline in all cognitive tests (β = −0.035 [95% CI −0.050 to−0.019] SD/y for global cognition; β = −0.047 [95% CI −0.068 to −0.025] SD/y for episodic memory; β = −0.019 [95% CI −0.034 to −0.004] SD/y for executive function)	
Nilaweera, 2022 [32], France	Longitudinal (14 years), 1562 participants aged ≥65, ESPRIT study cohort	25-item questionnaire to assess ACEs, using a modified version of the Childhood Trauma Questionnaire	Cognitive tests assessed global cognition, visual memory, verbal fluency, psychomotor speed (PS) and executive function. Incident dementia (Dx DSM-IV criteria)	At baseline, women having ≥5 ACEs (vs. 0–2) had poor psychomotor speed (OR = 1.52 95% CI 1.07–2.17). Also, participants with 3–4 ACEs (vs. 0–2) had worse verbal fluency (OR = 1.34 95% CI 1.00–1.78). Early abuse/maltreatment and poverty/financial difficulties were associated with worse PS.	No associations with incident dementia.
Stebbins, 2022 [33], USA	Longitudinal cohort, 7785 participants aged ≥65; HRS	Life course (ever have or been) traumatic events (TEs), e.g., child ever died? ever been in a major fire, flood, earthquake, or other natural disaster? ever have a life-threatening illness or accident? Before age 18: did your parents drink or use drugs? ever physically abused by parents?	Telephone Interview for Cognitive Status (HRS-TICS)	≥1 TEs over the life course was associated with accelerated cognitive decline (β = −0.05 95% CI: −0.07, −0.02) HRS-TICS units/year; 1 vs. 0 events) compared to experiencing no events.	Experiencing TEs was associated with better cognitive function cross-sectionally. No associations with incident dementia.
Voyer, 2023 [34], USA	Cross-sectional, 17,042 participants aged ≥45; 2020 BRFSS	Eleven questions for ACEs were converted to a summed ACEs score. As binary variable was used ≥2 ACEs vs. 0–1.	Subjective cognitive decline (SCD): had confusion or memory loss more often or is getting worse in the past 12 months	Having ≥ 2 ACEs (vs. 0–1) increased the odds of SCD (OR = 1.69 95% CI 1.36–2.10).	
Nilaweera, 2023 [35], Australia	Longitudinal 12,789 participants aged ≥70; ASPREE and ALSOP studies	Ten adverse life events related to interpersonal relationships (e.g., death of a spouse), finances (e.g., major money problems) and external factors (e.g., major accident or disaster).	Cognitive tests assessed global cognition, episodic memory, delayed recall, executive function, and psychomotor speed. Incident cognitive decline: those whose cognitive score dropped by >1.5 SD. Diagnosis of dementia—DSM-IV criteria.	Experiencing death of a spouse/partner (HR = 1.72 95% CI 1.17–2.52) and major financial problems (HR = 1.53 95% CI 1.05–2.23) were associated with increased risk of dementia. Experiencing financial problems was associated with increased risk cognitive decline in men (HR = 1.43 95% CI 1.10–1.86).	Women with some events (e.g., close family or friends lost their job/retired) had low risk of dementia (HR = 0.62 95% CI 0.40–0.95).
Zuelsdorff, 2024 [36], USA	Longitudinal cohort, 13,952 participants aged ≥55; HRS	Childhood traumatic events (CT): 4 items about childhood (e.g., before you were 18 years old, were you ever physically abused by parents?). 7 events at any age (e.g., Were you the victim of a serious physical attack or assault in your life?) and, if yes, the year that occurred. CT index (0–11).	27-item adapted Telephone Interview on Cognitive Status (TICS)	In White participants only (n = 11,607), greater childhood trauma exposure predicted worse baseline cognition (global cognition, immediate recall and delay recall) but slower change over time (global cognition and immediate recall).	
Hu, 2024 [37], UK	Longitudinal, cohort, mean age 55.9 (7.7) at baseline; UK Biobank (n = 150,152)	Childhood adversity (CA) (abuse or neglect; childhood trauma screen, 0–5)	Dementia (ICD-10 diagnosis)	Individuals with any CA had a greater risk for dementia (OR = 1.30, 95% CI 1.13–1.50) compared with those who did not experience CA	Depression, smoking, low grip strength and biomarkers were mediators.
Wang, 2025 [38], China	Longitudinal, 6178 participants aged ≥60; CHARLS	13 childhood adversities (before age 17), e.g., parental death, anxiety, depression, mental illness, disability, drug abuse, alcohol abuse, or involvement in criminal activities; a bedridden parent, lack of affection, neglect, physical abuse, domestic violence	Cognitive function evaluation comprises of an episodic memory test (immediate and delayed recall) and an attention test (total score 0–25)	Multiple childhood adversities were associated with lower cognitive function (β = −0.36, 95% CI [−0.58, −0.14]). Participants having rapid cognitive decline with moderate physical-mental deterioration, experiencing 2 childhood adversities predicted lower cognitive function (β = −0.88, 95% CI [−1.62, −0.14]).	
Assuras, 2025 [39], USA	Prospective cohort, n = 908 (matched control group, n = 667); New York	Documented physical, sexual abuse or neglect; 5 interviews from mean age 29.2 to 59.4	Comprehensive neuropsychological assessment battery (12 tests). Cognitive impairment with no dementia (CIND) or dementia	Maltreated individuals in both age groups were at increased risk for CIND [older (>age 59, OR = 2.08 95% CI 1.52–2.86) and younger (<age 59, OR = 1.63 95% CI 1.11–2.40)], compared to controls	Any maltreatment was associated with amnestic or non-amnestic CIND
Choi, 2025 [40], USA	Cohort of 8678 dementia-free participants aged ≥60 and <90 at baseline with data on APOE genotype; HRS	Retrospective early life conditions (deficits in financial, social, and human capital, as well as poor childhood health)	Dementia incidence (Langa–Weir Classification of Cognitive Function)	Low social capital (cause-specific HR = 1.24 95% CI 1.06–1.45), low human capital (HR = 1.46, 95% CI 1.29–1.66), and any health conditions (HR = 1.12 95% CI 1.01–1.25) were also associated with greater risk of dementia.	
Li, 2025 [41], UK	Cohort of 10,893 participants aged ≥50 at baseline; ELSA	Psychosocial stressors (financial strain, caregiving, disability, and limiting long-term illness)	Cognitive function: an overall global cognition score and domains scores (memory, executive function, and orientation)	Multiple stressors were associated with steeper declines in global cognition (B = −0.34, 95% CI [−0.45, −0.23], *p* < 0.001), memory (B = −0.12, 95% CI [−0.16, −0.07], *p* < 0.001), and executive function (B = −0.20, 95% CI [−0.29, −0.11], *p* < 0.001).	
Lian, 2025 [42], Puerto Rico	Longitudinal, cohort of, 3713 participants aged ≥60; PREHCO	Childhood adversity, 13 items (e.g., household SES, indicators of health status during early childhood and adolescence, neighborhood characteristics)	Mini-mental Cabán (MMC) (score 0–20) for global cognition. Cognitive impairment was defined as MMC scores 1.5 SD or more below the expected score at baseline	At baseline, parental illiteracy (β = −0.35, *p* < 0.001), neighborhood disadvantage (β = −0.27, *p* < 0.001), economic hardship (β = −0.10, *p* = 0.003) and childhood illness (β = −0.21, *p* < 0.001) were associated with MMC scores. Neighborhood disadvantage was associated with incident cognitive impairment (OR = 1.19 95% CI 1.06–1.34, *p* = 0.003).	Depression and self-rated health were mediators
Liu, 2025 [43], USA	Longitudinal, cohort of, 51,327 participants aged ≥50; HRS	ACEs include 6 questions from two dimensions: financial adversity and traumatic events (total score 0–6)	Dementia was evaluated using the modified Telephone Interview for Cognitive Status (TICS-m)	ACEs were associated with increased risk of dementia (HR = 1.08 95% CI 1.02–1.16)	The association of ACEs and dementia was fully mediated by early life stage cognitive reserve enhancing factor and partially mediated by adulthood enhancing factor

Only significant (*p* < 0.05) multivariate results are reported in the table. OR = odds ratios; HR = hazard ratios; B = unstandardized beta estimated; CI = confidence intervals; SD = standard deviation; ACEs = Adverse Childhood Experiences; AD = Alzheimer’s Disease. KIHD = Kuopio Ischemic Heart Disease Risk Factor; SHARE = Survey of Health, Ageing, and Retirement in Europe; JAGES = Japan Gerontological Evaluation Study; Sydney MAS = Sydney Memory and Ageing Study; CHARLS = China Health and Retirement Longitudinal Study; ESPRIT = “Enquete de Sante’ Psychologique-Risques, Incidence et Traitement”. KHANDLE = Kaiser Healthy Aging and Diverse Life Experiences; ASPREE = ASPirin in Reducing Events in the Elderly study; ALSOP = ASPREE Longitudinal Study of Older Persons sub-study; HRS = US Health and Retirement Study; BRFSS = Behavioral Risk Factor Surveillance System; ELSA = English Longitudinal Study of Ageing; PREHCO = Puerto Rican Elder: Health Conditions.

**Table 2:** Studies related to Frailty.

Authors	Design, Population	Exposures	Geriatric Syndromes or Other Outcomes	Main Outcome’s Findings	Additional Comments
Haapanen, 2018 [44], Finland	Cases (n = 117; separated age 72.8 SD = 2.6) controls (n = 855 non-separated 70.5 SD = 2.5); HBCS	Early life stress (Wartime parental separation–World War II)	Frailty at a mean age of 71 years (Fried’s criteria: weight loss, low p. activity, exhaustion, weakness, and slowness between 2011 and 2013)	Compared to the non-separated men, men who had been separated had an increased frailty (RRR = 5.18 95% CI 1.16–23.17 *p* = 0.031).	No associations were observed among women
dos Santos Gomes, 2018 [45], Albania, Brazil, Canada, Colombia	Cross-sectional 2002 participants aged 65–74; IMIAS	Physical abuse during the first 15 years of life. Adulthood domestic physical and psychological violence by family and intimate partners was assessed by the Hurt, Insult, Threaten and Scream (HITS) scale.	Frailty (Fried’s phenotype)	Childhood physical abuse (OR = 1.68; 95% CI 1.01–2.78) and psychological violence by their intimate partner (OR = 2.07 95% CI 1.37–3.12) were associated with frailty.	
Van Der Linden, 2020 [46]; Switzerland	Longitudinal cohort 23,358 participants aged ≥50; SHARE	ACEs score 0–7: e.g., child in foster care, parental (death, mental illness, or drinking abuse), period of hunger, and property taken away (≥1 vs. 0). Adverse childhood health experiences (ACHE) e.g., serious health conditions, long hospitalization, (≥1 vs. 0)	Frailty (Fried’s phenotype): weakness, shrinking, exhaustion, slowness, or low activity.	Participants having at least one ACE (OR = 1.30 95% CI 1.14–1.48 *p* < 0.001) or one ACHE (OR = 1.40 95% CI 1.24–1.57 *p* < 0.001) had increased odds for frailty.	
Li, 2020 [47]; China	Longitudinal cohort 6806 participants aged ≥60; CHARLS	Early life risk factors (childhood or adolescence): starvation, domestic violence, neighborhood quality [0 (lowest) to 4 (highest)], childhood health status (e.g., healthier vs. same as others)	Frailty: slowness, weakness, exhaustion, inactivity and weight loss (robust, prefrail, frail)	Better childhood neighborhood quality (e.g., 4 vs. 0, OR = 0.28 95% CI 0.15–0.52) and better childhood health status (OR = 0.74 95% CI 0.57–0.96) had lower risk of being frail. Childhood severe starvation was associated with higher risk of prefrailty (OR = 1.30 95% CI 1.04–1.62)	
Ye, 2021 [48]; China	Longitudinal cohort 11,615 individuals aged ≥45; CHARLS	Childhood food deprivation (CFD): not enough food to eat before age 12, or born and brought up in famine affected areas and in famine periods in China.	FRAIL scale as a sum of fatigue, resistance, ambulation, illness, and loss of weight (robust, prefrail, frail)	CFD was associated with frailty at old age (OR = 1.30 95% CI 1.26–1.36). Those with extreme CFD (vs. mild CFD) had increased risks of frailty (OR = 1.34 95% CI 1.26–1.43).	
Baranyi, 2022 [49], UK	Longitudinal cohort, 363 participants, mean age 69.3 (SD = 0.74); LBC1936	Neighborhood social deprivation (NSD): population density, overcrowding, infant mortality, households renting; male unemployment, overcrowding, car ownership, and social class. During childhood, young adulthood, late adulthood	Frailty Index (FI) 30 deficits covering physical, psychological, and cognitive systems	Among males, greater accumulated NSD was associated with higher FI at baseline (B = 0.017, 95% CI 0.005, 0.029). Among females, in late adulthood, higher NSD was associated with widening frailty trajectories (B = 0.005, 95% CI 0.0004, 0.009).	
Yan, 2022 [50], China	Longitudinal cohort 10,963 participants aged ≥45; CHARLS	ACEs (before age 17) with 17 indicators criteria; including abuse and neglect, death, illness/disability, living environment outside the home -score 0 to 1, with greater values indicating severe ACEs), childhood SES, and childhood health and health care	Frailty Index (32 deficits in six domains were chosen to construct FI, score 0 to 1)	ACEs, childhood SES, and indicators of childhood health and health care were associated with baseline and change rate of FI. ACEs (B = 0.018 [SE = 0.004], *p* < 0.001), self-rated childhood SES (−0.001 [0.000], *p* < 0.01), and objective health and health care (−0.002 [0.001], *p* < 0.05) were significantly associated with the slope (latent growth curve, FI trajectory).	
Haapanen, 2022 [51], Finland	Longitudinal cohort 2000 participants aged 57–84 years (3 waves over 17 years); HBCS	Early life stress (Wartime parental separation–World War II)	Frailty Index (FI) assessed with 41 deficits, at a mean age of 57 years.	Women separated from their parents during War had steeper increase in FI levels percentage point differences of change per year from midlife to old age (B = 0.211 95% CI 0.009, 0.414; *p* = 0.041)	No associations were observed among men
Mian, 2022 [52], Canada	Cross-sectional, 23,354 people aged 45–85 years; CLSA	ACEs (8-item) (3 abuse types; neglect; parental divorce, mental illness or death; intimate partner violence)	Frailty Index (76-item FI) (based on Rockwood and Mitnitski)	ACEs were associated with FI (≥3 ACEs vs. 0, B = 0.04 [95% CI 0.037, 0.044])	
Gao, 2022 [53], China	Cross-sectional, 7342 participants aged ≥65; CLHLS	Childhood hunger (CH) “Did you often go to bed hungry as a child?” “yes” or “no.”	Frailty Index (FI) using 44 health deficits, including daily life events, chronic illness, and psychological functioning	Childhood hunger was associated with frailty among participants 65–79 years (OR = 1.21 95% CI 1.03–1.43),	CH at aged ≥80 years had lower odds of frailty (OR = 0.80 95% CI 0.65–0.98).
Wang, 2022 [54], China	Longitudinal, 11,568 participants aged ≥45, CHARLS	ACEs covering childhood intrafamilial aggression, family dynamics, SE deprivation, loss or threat of loss within the family, and neighborhood quality (score 0–18)	Frailty Index (FI) using 41 measures. Robust, prefrail, and frail, with its trajectories classified as stable at robust and prefrail and rapidly rising to frail.	An increased number of ACEs was associated with a frail status (OR = 1.20 95% CI 1.16–1.23) and being in the rapidly rising trajectory (OR = 1.19 95% CI 1.16–1.23).	
Dimitriadis, 2023 [55], the Netherlands	Baseline (n = 2176) aged 58–89, longitudinal 17-yr follow-up (n = 1427); LASA	ACEs: War experiences, a parent death, excessive alcohol use of a relative, sexual abuse, severe problems at home, parents’ poverty, physical illness of respondent	LASA-FI with 32 deficits (score 0.0 to 0.7; Frailty is defined as a FI ≥ 0.25)	ACE and frailty were associated at baseline (OR = 1.88 95% CI 1.46–2.42; *p* < 0.001) and at follow-up (HR = 1.28 95% CI 1.01–1.64; *p* = 0.044)	
Wang, 2023 [56], China	Longitudinal 43,928 participants from Europe (aged ≥50) and China (aged ≥45); SHARE and CHARLS	ACEs covering childhood intrafamilial aggression, family dynamics, SE deprivation, loss or threat of loss within the family, and neighborhood quality (score 0–18)	Frailty Index (FI) using 35 attributes (score 0 to 1). Robust (FI ≤ 0.10), prefrail (FI > 0.10 and <0.25), and frail (FI ≥ 0.25). Trajectories: stable at robust and prefrail, rapidly increasing to frail.	ACEs > 3 (vs. ≤1) was associated with a frail status for men (OR = 1.69 95% CI 1.36–2.09) and women (OR = 2.38 95% CI 1.93–2.94). ACEs > 3 (vs. ≤1) was associated with FI trajectory of rapidly rising to frail for men (OR = 1.70 95% CI 1.24–2.34) and women (OR = 2.12 95% CI 1.70–2.63).	
Yang, 2024 [57], UK	Cross-sectional, 152,914 adults aged 40–69; from the UK Biobank	Childhood adversity 5-item: physical neglect, emotional neglect, sexual abuse, physical abuse, and emotional abuse	Frailty Index (FI) using 49 items covering sensory, cranial, mental well-being, infirmity, cardiometabolic, musculoskeletal, immunological, cancer, pain, and gastrointestinal. A frailty score (0 to 1)	Childhood adversity was associated with frailty (OR = 1.38 95% CI 1.36–1.40; *p* < 0.001)	Unhealthy lifestyle score (0–5; (calculated based on BMI, smoking, alcohol, activity, and diet) was a mediator
Xian, 2025 [58], China	Cross-sectional (2017–2018), 4473 participants with mean age 60.0 ± 5.4; CHARLS	Famine fetal exposed (1959–1962), preschool exposed (1954–1957), and school-aged (1950–1953), compared to the non-exposed group (1964–1967).	Frailty Index (FI) based on 32 items for disability, comorbidity, physical functioning, cognitive impairment, and depressive symptoms. Robustness (FI ≤ 0.10), prefrailty (FI > 0.10 and <0.25), and frailty (FI ≥ 0.25)	There was a significant increase in the probability of frailty compared to the non-exposed group in fetal (OR = 2.84 95% CI 1.73–4.65) and school-age (OR = 2.81 95% CI 1.73–4.57) exposed groups.	

Only significant (*p* < 0.05) multivariate results are reported in the table. OR = odds ratios; RRR = relative risk ratio; B = unstandardized beta estimated; SE = standard error; CI = confidence intervals; SD = standard deviation; BMI = body mass index; ACEs = Adverse Childhood Experiences. HBCS = Helsinki Birth Cohort Study; IMIAS = International Mobility in Aging Study; SHARE = Survey of Health, Ageing, and Retirement in Europe; CHARLS = China Health and Retirement Longitudinal Study; LBC1936 = Lothian Birth Cohort 1936; CLSA = Canadian Longitudinal Study on Aging; CLHLS = Chinese Longitudinal Healthy Longevity Survey; LASA = Longitudinal Aging Study Amsterdam.

**Table 3:** Studies related to Depression.

Authors	Design, Population	Exposures	Geriatric Syndromes or Other Outcomes	Main Outcome’s Findings	Additional Comments
Ege, 2015 [59], USA	Cross-sectional, 8051 participants aged ≥60; 2010 BRFSS data	ACEs: parents being physically abusive to each other, being physically harmed by a parent, being sworn at by the parent, being touched sexually by an adult, being forced to sexually touch an adult, and being forced into a sexual encounter	Depressive symptoms 8-item the Patient Health Questionnaire (PHQ-8). Depression a score of ≥10.	A single occurrence of forced sexual intercourse was associated with late-life depression (OR = 2.92 95% CI 1.06–8.02). Repeated physical abuse and repeated forced sexual intercourse were also associated with depression (OR = 2.94 95% CI 1.68–5.13; OR = 3.66 95% CI 1.01–13.2, respectively).	
Cheong, 2017 [60]; Ireland	Cross-sectional, 2047 participants aged 50–69; Livinghealth Clinic Mitchelstown (Ireland) 2010–2011 cohort	ACEs (0–10), three domains: abuse, neglect, household dysfunction. Perceived social support (Oslo Social Support Scale), scores 3–8 (poor), 9–11 (moderate) and 12–14 (strong)	CES-D scale (0–60) ≥16 was defined as having depressive symptoms	Among individuals reporting poor perceived social support (PSS), having any ACE (vs. 0) was associated with depressive symptoms (OR = 2.85 95% CI 1.64–4.95). Among those reporting moderate PSS, the odds were OR = 2.21 95% CI 1.52–3.22	
Flores, 2018 [61], USA	Cross-sectional, 2000 participants aged ≥60; SABE Bogota	History of sexual abuse (ever being a victim)	Using the GDS (score 0–15), depression was defined as score ≥ 6	Higher odds of depression for past sexual abuse (OR = 3.91 95% CI 2.13–7.16)	Being displaced by violence was also associated with depression
Iob 2020 [62]; England	Longitudinal, 4382 participants aged ≥50; ELSA	ACEs before age 16, with 4 dimensions: threat (e.g., abuse), household dysfunction (e.g., parent mental illness or substance abuse), low parental bonding (maternal and paternal), and loss experiences (e.g., parent death, or foster care).	Depressive symptoms were assessed using the 8-item Centre for Epidemiological Studies Depression scale (CES-D-8) (score 0–8)	Greater ACEs cumulative exposure was associated with higher depressive symptoms at baseline (β = 0.149 95% CI 0.115–0.183) and with their increase over time (β = 0.355 95% CI 0.184–0.526).	Higher baseline CRP levels mediated ACEs cumulative exposure on the baseline value and change in depressive symptoms.
Yazawa, 2022 [63]; Japan	Cohort, 7271 participants aged ≥65; JAGES	ACEs, having ≥ 2 experiences: parental loss, parental divorce, parental mental illness, domestic violence, physical abuse, psychological neglect or abuse, and economic disadvantage	Depressive symptoms were assessed using the GDS-15. Score 0–15; or ≥5 mild-to-severe depressive symptoms	ACEs was associated with greater depressive symptoms after adjusting for low adult SES (B = 0.41 95% CI 0.25–0.57).	Adult SES was a mediator
Guo, 2023 [64]; China	Cross-sectional, 11,452 participants aged ≥45, CHARLS	ACEs 12 items that occurred before the age of 17 years (score 0–12)	Depressive symptoms were measured by the CES-D-10 (Epidemiologic Studies Depression Scale) (score 0–30). Cutoff point of 10 for clinically significant levels	Having ≥4 ACEs (vs. 0) was associated with depressive symptoms OR = 3.38 95% CI 2.92–3.90)	Short sleep duration was a mediator
Li, 2023 [65]; China	Cross-sectional, 1091 participants aged ≥60, in Jinan, China.	Childhood traumatic events as the number of traumatic events (0–15) before age 18. Their impact on their lives (0 = not at all to 4 = very hard; total score 0–60 points for childhood trauma severity [CTS]).	Depressive symptoms were assessed using the GDS-15 (Score 0–15)	Childhood traumatic events (β = 0.131 (SE = 0.041) 95% CI = 0.048–0.209, *p* = 0.003), childhood trauma severity (β = 0.109 (SE = 0.033) 95% CI = 0.043–0.171, *p* = 0.002).	Resilience mediated the relationship between CTS and depressive symptoms
Dai, 2024 [66], China	Longitudinal; 6921 participants aged ≥45; CHARLS	ACEs (10- item) 5 threats (e.g., physical abuse) and 5 deprivations (e.g., emotional neglect).	Depressive symptoms (CES-D-10 scale) trajectory	Compared to individuals without ACEs, those with ≥4 ACEs had a significantly higher odds of following the continuing-high trajectory (OR = 20.22 95% CI 12.11–33.74), rather than the continuing-low trajectory	Mediators were arthritis, digestive, and respiratory diseases
Babatunde, 2024 [67]; USA	Cross-sectional, 60,122 participants aged ≥60; 2020 BRFSS	ACE score (0, 1, 2–3, and ≥4) included exposure to eight types of ACEs before age 18	Self-reported history of depression (health professional diagnosis)	Experiencing ≥4 ACEs (vs. 0) had higher odds for depression among Whites (OR = 3.83 95% CI 3.07–4.79), Blacks (OR = 3.39 95% CI 1.71–6.71), and Hispanics (OR = 12.61 95% CI 4.75–33.43).	
Jiang, 2025 [68]; China	Longitudinal, 3941 participants aged ≥45; CHARLS	ACEs 10 items before the age of 18 (e.g., parental divorce, childhood hunger). Five items for Adverse Adult Experiences (AAEs) (e.g., death of a child, lifetime discrimination)	Depressive symptoms (DS) were assessed using the 10-item Center for Epidemiologic Studies Depression Scale (CES-D 10) (score 0–30). A cutoff score > 10 indicated probable depression	Six ACEs were associated with DS [e.g., childhood hunger (OR = 1.23 95% CI 1.03–1.47), dangerous growth environments (OR = 1.34 95% CI 1.09–1.65)] and 2 AAEs like prolonged bed rest (OR = 1.39 95% CI 1.08–1.79), and lifetime discrimination (OR = 1.37 95% CI 1.12–1.66)	Arthritis or rheumatism was a partial mediator
Rhee, 2025 [69]; USA	Cross-sectional, 315 Korean Americans residing in affordable senior housing.	ACEs 10 items before the age of 18, such as maltreatment (e.g., neglect, physical, verbal, or sexual abuse) and household dysfunction (e.g., parental separation/incarceration, substance use, or mental health issues).	Depressive symptoms (DS) using the Patient Health Questionnaire-9 (PHQ-9)	ACEs were associated with DS in multivariate analysis (B = 0.84 SE = 0.18, *p* < 0.001); adding potential protective factors (B = 0.68 SE = 0.17, *p* < 0.001) and interactions (B = 0.46 SE = 0.19, *p* < 0.05)	
Li, 2025 [70]; China	Cross-sectional, 6585 participants aged ≥60; CHARLS	ACEs (range 0–12): 7 conventional (physical abuse, emotional neglect, household substance abuse, mental illness, domestic violence, incarcerated household member, parental separation/divorce); 2 expanded (unsafe neighborhood, bullying); and 3 novel indicators (parental death, sibling death, parental disability)	Depression via the CES-D-10 (cutoff ≥ 12).	ACEs was associated to depression (OR = 1.17 95% CI 1.11–1.24).	Mediators were sleep duration and chronic diseases
Dai, 2025 [71], China	Longitudinal; 6395 participants aged ≥60; CHARLS	Poor childhood friendship experiences (CFE) (self-report)	Depressive symptoms (CES-D-10 scale, score 0–30); ≥10 significant depressive symptoms	Individuals with poor CFE had a greater risk for depressive symptoms (OR = 1.64, 95% CI 1.22–2.21) compared with those with better CFE	
Chen, 2026 [72]; China	Longitudinal, 3734 participants aged ≥45; CHARLS	ACEs score 0–10; (e.g., adverse peer relationships, emotional abuse, domestic violence, household substance abuse or incarceration, parental mental illness, family discord, neglect, family member death, family burden, and adverse outside-family environment.	Depressive symptoms (DS)- Center for Epidemiologic Studies Depression Scale (CES-D 10) score 0–30, ≥10 significant depressive symptomatology	Having ≥4 ACEs was associated with DS trajectories, stable increasing vs. low-stable type (OR = 2.43 95% CI 1.78–3.32, *p* < 0.001). For each one-point increase in ACE scores, there is an increase of 0.501 (95% CI 0.430–0.570, *p* < 0.001) CES-D score.	

Only significant (*p* < 0.05) multivariate results are reported in the table. OR = odds ratios; HR = hazard ratios; B = unstandardized beta estimated; β = standardized beta estimated; CI = confidence intervals; BMI = body mass index; ACEs = Adverse Childhood Experiences. CES-D-10 = Center for Epidemiologic Studies Depression Scale. GDS = Geriatric Depression Scale. BRFSS = Behavioral Risk Factor Surveillance System; SABE = Health, Well-being, and Aging; ELSA = English Longitudinal Study of Ageing; CHARLS = China Health and Retirement Longitudinal Study; JAGES = Japan Gerontological Evaluation Study.

**Table 4:** Studies related to Falls.

Authors	Design, Population	Exposures	Geriatric Syndromes or Other Outcomes	Main Outcome’s Findings	Additional Comments
Reyes-Ortiz, 2018 [73]; USA	Cross-sectional, 2000 participants aged ≥60; SABE Bogota	History of abuse (ever being a victim): emotional, physical, or sexual. Polyvictimization (≥2 types of abuse vs. 0–1)	Falls during past 12 months. Any fall (≥1 vs. 0) or recurrent falls (≥2 vs. 0–1)	Abuse types: emotional (OR = 1.43 95% CI 1.07–1.91), physical (OR = 2.05 95% CI 1.51–2.77), or sexual (OR = 2.11 95% CI 1.12–3.99), and polyvictimization (OR = 2.39 95% CI 1.72–3.33) were associated with recurrent falls.	
Reyes-Ortiz, 2021 [74]; USA	Cross-sectional, 5227 participants aged ≥60; SABE Ecuador	History of abuse (ever being a victim): physical, emotional, sexual, financial, or caregiver neglect. Polyvictimization (≥2 types of abuse vs. 0–1)	Falls during past 12 months. Recurrent falls (≥2 vs. 0–1)	Polyvictimization was associated with higher odds of recurrent falling (OR = 1.45 95% CI 1.20–1.76)	Depressive symptoms were a mediator
Reyes-Ortiz, 2022 [75]; USA	Cross-sectional, 19,004 participants aged ≥60; SABE Colombia	Everyday discrimination (ever had discrimination e.g., racial, SES, gender, age, religion, sexual orientation, or disability), childhood discrimination score (0–3), and discrimination last 5 years due to skin color (score 0–4; at group activities, public places, family, or health centers)	Falls during past 12 months. Recurrent falls (≥2 vs. 0–1)	Everyday discrimination (OR = 1.27 95% CI 1.21–1.33), childhood discrimination score (OR = 1.23 95% CI 1.13–1.33), and number of situations for discrimination (OR = 1.12 95% CI 1.08–1.17) were associated with recurrent falling.	
Reyes-Ortiz, 2024 [76]; USA	Cross-sectional, 18,875 participants aged ≥60; SABE Colombia	History of age discrimination or ageism by (1) the neighborhood (2) family (3) health services (4) public services	Falls during past 12 months. Recurrent falls (≥2 vs. 0–1).	Any ageism (OR = 1.81 95% CI 1.61–2.02, *p* < 0.0001) was associated with recurrent falling.	Mediators were depressive symptoms, low IADL, and multimorbidity
Huang, 2024 [77], China	Longitudinal; 12,061 participants aged ≥45, CHARLS	ACEs (12- item, self-report) (physical abuse, childhood environment and adversity [e.g., health status, death of parents, neighborhood safety, hunger, bullying]	Falls since last survey	Those with ≥ 5 ACEs vs. ≤2 ACEs (OR = 1.85 95% CI 1.65–2.08) were more likely to experience falls; higher number of ACEs (OR = 1.16 95% CI 1.13–1.19) was associated with higher risk of fall.	Depressive symptoms were a mediator
Tan, 2025 [78], China	Cross-sectional; 14,582 middle-aged, older adults, CHARLS	ACEs (12- item, self-report) (e.g., physical abuse, emotional neglect, household dysfunction, parental divorce or death, bullying)	Falls since last survey	ACEs were associated with falls (≥4 ACEs vs. 0; OR = 2.56 95% CI 2.12–3.09, *p* < 0.001)	Pain and depressive symptoms were mediators
Reyes-Ortiz, 2025 [79], USA	Cross-sectional; 38,437 participants age 45 to 80, BRFSS	ACEs (11- item, self-report) abuse domain (5 questions) and household dysfunction domain (6 questions). Polyvictimization (≥2 types of abuse) and poly-dysfunction (≥2 types of household dysfunction) were also assessed.	Falls during past 12 months (Any falls: ≥1 vs. 0) among middle-aged (45–64 years) and older adults (≥65)	Those with ≥2 ACEs (vs. 0–1) have increased odds of falling among middle-aged (OR = 1.34 95% CI 1.18–1.52) and older adults (OR = 1.28 95% CI 1.15–1.43). Polyvictimization and poly-dysfunction were also associated with falling in both age groups	Mediators: depression, functional difficulties, multimorbidity and difficulty remembering

Only significant (*p* < 0.05) multivariate results are reported in the table. SES = socioeconomic status. ACEs = Adverse Childhood Experiences. OR = odds ratios; CI = confidence intervals. IADL = instrumental activities of daily living. SABE = Health, Well-being, and Aging; CHARLS = China Health and Retirement Longitudinal Study; BRFSS = Behavioral Risk Factor Surveillance System.

**Table 5:** Studies related to Low Muscle Strength or Sarcopenia.

Authors	Design, Population	Exposures	Geriatric Syndromes or Other Outcomes	Main Outcome’s Findings	Additional Comments
Cheval, 2018 [80], Switzerland	Cohort, 24,179 aged 50–96, SHARE	Early life SEC (socioeconomic circumstances) at age 10; based on 4 SEC indicators, 5-level variable from most disadvantaged to most advantaged.	Handgrip strength, defined low muscle strength (LMS) (cutoff obtained after being stratified by gender and BMI quartiles)	Among women early life SEC disadvantaged (OR = 0.79 95% CI 0.64–0.99), middle (OR = 0.69 95% CI 0.54–0.86), and advantaged (OR = 0.58 95% CI 0.44–0.77) had lower odds for low muscle strength compared to those most disadvantaged.	Compared to those most disadvantaged men, disadvantaged (OR = 0.67 95% CI 0.51–0.88), and middle (OR = 0.74 95% CI 0.56–0.98) had lower odds for LMS
Cheval, 2019 [81], Switzerland	Cohort, 24,179 aged 50–96, SHARE	ACEs (child in care, parental death, parental mental illness, parental drinking, period of hunger, or property taken away)	Handgrip strength, defined low muscle strength (LMS) (cutoff obtained after being stratified by gender and BMI quartiles)	For women, there was a gradual increase in the risk of LMS with the number of experienced ACEs (OR = 1.22 for 1 ACE, OR = 1.74 for ≥2 ACEs compared to no ACE)	No significant association among men.
Smith, 2019 [82], USA	Cohort, 4459 aged ≥50, HRS	Childhood misfortune (conditions experienced before age 18) 5 domains: infectious disease, chronic disease, impairment, SES, and risky parental behavior	Handgrip strength (HGS) in Kg	For men, at follow-up (Time 2), there was a steeper decline in HGS (B = −0.60, *p* < 0.05) when having one childhood impairment	At follow-up, no significant association among women.
Lin, 2022 [83], China	Cross-sectional, 7209 participants aged ≥45, CHARLS	ACEs before age 17: physical abuse, emotional neglect, household substance abuse, household mental illness, domestic violence, incarcerated household member, parental separation or divorce, unsafe neighborhood, bullying, parental death, sibling death, and parental disability	Handgrip strength (HGS) in kilograms (continuous); low muscle strength (LMS; defined based in sex and BMI)	Having ≥ 3 ACEs (vs. 0) was negatively associated with lower HGS in Kg (*β* = −0.93, 95% CI −1.37, −0.49) and positively associated with having LMS (OR = 1.34 95% CI 1.12–1.61).	
Huang, 2024 [84], China	Longitudinal, 6859 participants aged ≥45; CHARLS	ACEs, two adversities domains, 5 threat-related (physical abuse, household substance abuse, domestic violence, unsafe neighborhood, or bullying), and 5 deprivation related (emotional neglect, household mental illness or incarceration, parental death).	Sarcopenia (Asian Working Group for Sarcopenia 2019 algorithm), was diagnosed when low muscle mass, and low muscle strength or poor physical performance were detected	ACEs were associated with increased risk for sarcopenia: ≥3 ACEs (vs. 0) (HR = 1.31 95% CI 1.10–1.56); participants with ≥2 threat-related ACEs (vs. 0) (HR = 1.22 95% CI 1.04–1.43) or with ≥2 deprivation-related ACEs (vs. 0) (HR = 1.22 95% CI 1.02–1.46)	Active social participation modified the association between ACEs, (especially threat-related), and sarcopenia
Dimitriadis, 2025 [85], Canada	Cross-sectional, 25,327 participants aged 45–85 years; CLSA	ACEs: War experiences, a parent death, excessive alcohol use of a relative, sexual abuse, severe problems at home, parents’ poverty, physical illness of respondent	Sarcopenia was defined using the revised EWGSOP2 guidelines	Significant protective association for ACEs (Two ACEs vs. No ACE) on sarcopenia emerged in the oldest group (75–85 years; OR = 0.72 95% CI 0.54–0.95 *p* = 0.021)	Findings might be explained by bias due to survival of the fittest

Only significant (*p* < 0.05) multivariate results are reported in the table. OR = odds ratios; HR = hazard ratios; B = unstandardized beta estimated; β = standardized beta estimated; CI = confidence intervals; BMI = body mass index; ACEs = Adverse Childhood Experiences. SHARE = Survey of Health, Ageing, and Retirement in Europe; HRS = Health and Retirement Study; CHARLS = China Health and Retirement Longitudinal Study; CLSA = Canadian Longitudinal Study on Aging; EWGSOP2 = European Working Group of Sarcopenia in Older People.

**Table 6:** Studies related to Multimorbidity.

Authors	Design, Population	Exposures	Geriatric Syndromes or Other Outcomes	Main Outcome’s Findings	Additional Comments
Vásquez, 2019 [86], USA	Cross-sectional, 10,727 participants aged ≥55, 2012-13 NESARC-III	Before age 18 (a) maltreatment (#5): physical, emotional/sexual abuse; physical/emotional neglect; (b) others (#11): i.e., witnessing domestic violence; parents divorced, parents died; (c) past year stressful life events (#9), i.e., unemployed, bankruptcy. ACEs defined any maltreatment or other adverse events (1 = Yes, 0 = No)	Multimorbidity based on # of somatic conditions (i.e., heart disease, hypertension, stroke, diabetes, arthritis, cancer, osteoporosis and chronic lung problems) and DSM-5 psychiatric disorders (i.e., depression) during past 12 months, adjusted means ± SE	History of ACEs, compared with no history, had greater numbers of somatic and psychiatric multimorbidity (1.37 ± 0.04 vs. 1.13 ± 0.04) among racial and ethnic middle-aged adults (55–64)	No association among older adults (≥65)
Lin, 2021 [87], China	Cross-sectional, 11,972 participants aged ≥45, CHARLS	ACEs (0–12), 7 conventional (physical abuse, emotional neglect, household substance abuse, household mental illness, domestic violence, incarcerated household member, and parental separation or divorce), 2 expanded (unsafe neighborhood and bullying), and 3 others (parental death, sibling death, and parental disability)	Multimorbidity, defined as ≥2 of 14 chronic diseases.	Having ≥4 ACEs (vs. 0) was associated with higher odds for multimorbidity (OR = 2.03 95% CI 1.70–2.41). In addition, using only the 7 conventional ACEs, having ≥ 4 ACEs (vs. 0) was also associated with multimorbidity (OR = 2.52 95% CI 1.81–3.50).	
Reyes-Ortiz, 2023 [88]; USA	Cross-sectional, 18,873 participants aged ≥60; SABE Colombia	Three racial discrimination measures: (1) everyday discrimination (ever had), (2) childhood discrimination score (0–3), and (3) situations of discrimination in the last 5 years (score 0–4 [group activities, public places, inside the family, health centers]).	Multimorbidity, defined as having 2 or more chronic conditions	Multimorbidity was associated with experiencing everyday discrimination (OR = 2.21 95% CI 1.62–3.02), childhood discrimination (OR = 1.27 95% CI 1.10–1.47), and the number of situations of discrimination (OR = 1.56 95% CI 1.22–2.00).	
Chandrasekar, 2023 [89], UK	Longitudinal, 3264 cohort members, ages 36-, 43-, 53-, 63-, and 69- years. NSHD	Nine ACEs in 3 groups: (a) psychosocial, (b) parental health, and (c) childhood health.	Multimorbidity, based on 18 health disorders, its score was defined as the unweighted sum of disorders accumulated by an individual at each assessment	Combined childhood health ACEs were associated with higher and progressively increasing multimorbidity scores across adulthood (from 0.18 at age 36 to 0.45 at age 69).	Psychosocial ACE accumulation was associated with steeper multimorbidity trajectories at follow-up
Liu, 2024 [90], China	Longitudinal cohort, 6428 participants aged ≥45, CHARLS	ACEs: physical abuse, emotional neglect, household substance abuse, household mental illness, domestic violence, incarcerated household member, parental separation or divorce, unsafe neighborhood, bullying, parental death, sibling death, and parental disability	Multimorbidity was the number of physician diagnoses of 14 chronic diseases, the Chinese multimorbidity-weighted index (CMWI)	ACEs were associated with an increased number of chronic diseases at the baseline (intercept = 0.28, 95% CI 0.20–0.36) and a more rapid increase in the number of chronic diseases over 7 years (intercept = 0.03, 95% CI 0.01–0.05).	

Only significant (*p* < 0.05) multivariate results are reported in the table. OR = odds ratios; CI = confidence intervals; ACEs = Adverse Childhood Experiences. NESARC = National Epidemiologic Survey on Alcohol and Related Conditions; CHARLS = China Health and Retirement Longitudinal Study; SABE = Health, Well-being, and Aging. NSHD = National Survey of Health and Development.

**Table 7:** Studies related to Functional Decline.

Authors	Design, Population	Exposures	Geriatric Syndromes or Other Outcomes	Main Outcome’s Findings	Additional Comments
Guedes, 2016 [91], Albania, Brazil, Canada, Colombia	Cross-sectional, 1995 participants aged 65–74, IMIAS	Childhood physical abuse (CPA) (first 15 years of life). Adulthood domestic violence as intimate partner/family member violence (physical or psychological) (PA = physical abuse)	Lower extremity functioning measured by the SPPB = Short Physical Performance Battery including standing balance, 4-m walk, and chair stand test. Mobility disability was defined as SPPB < 8 or self-reported limitation in walking/climbing stairs.	CPA (OR = 1.69 95% CI 1.14–2.51), partner PA (OR = 1.78 95% CI 1.15–2.28) and family PA (OR = 1.62 95% CI 1.16–2.27) were associated with SPPB < 8. CPA (OR = 1.43 95% CI 1.03–1.99) and family PA (OR = 1.39 95% CI 1.06–1.83) were associated with mobility disability.	Chronic conditions and depression were mediators between physical violence and both mobility measures.
Amemiya, 2018 [92], Japan	Cross-sectional 19,220 participants aged ≥65; JAGES	ACEs before age of 18 including seven adversities: parental death, parental divorce, parental mental illness, family violence, physical abuse, psychological neglect, and psychological abuse	Higher-level functional limitations (HLFL), using the Lawton IADLs scale, 13 items with instrumental self-maintenance (5 items), intellectual activities (4 items), and social role (4 items).	ACEs (1 vs. 0) (PR = 1.06 95% CI = 1.02–1.10) and ACEs (≥ 2 vs. 0) (PR = 1.19 95% CI = 1.12–1.27). were associated with HLFL	Socio-demographics, adult health behaviors, and health status were mediators
Li, 2022 [93], China	Longitudinal, 10,651 participants aged ≥45, CHARLS	ACEs: 13 items including physical abuse, emotional neglect, domestic violence, household substance abuse, household mental illness, incarcerated household member, parental separation or divorce, parental death, sibling death, parental disability, bullying, peer rejection, and unsafe neighborhood.	ADLs disability, having difficulty or could not perform the activity. A 6-item summary ADLs, score 0–6.	Participants who had ≥4 ACEs had increased risk for being on low-mild trajectory (OR = 1.32 95% CI 1.11–1.57) and mild-increasing trajectory (OR = 1.41 95% CI 1.06–1.89), compared with those with 0 ACEs.	Chronic diseases were mediators: arthritis, digestive system disease, respiratory disease, and cardiometabolic disease.
Lee, 2023 [94], USA	Cross-sectional, 3387 participants aged ≥50, NSHAP	ACEs = childhood experience of violence/abuse, witnessing violence, financial insecurity, parental separation, or serious illness (exposures between ages 6 to 16)	Lower extremity functioning (tandem balance, 3-m walk and chair stand tests); physical mobility impairment was defined if those tests fell below clinical standards. Also, cognitive testing (MoCA) and reported functional disability (difficulty with ADLs or IADLs).	Childhood experience of violence was associated with greater physical mobility impairment (OR = 1.38, 95% CI 1.11–1.71) and functional disability (OR = 1.86, 95% CI 1.49–2.33).	

Only significant (*p* < 0.05) multivariate results are reported in the table. OR = odds ratios; PR = prevalence ratios; CI = confidence intervals ACEs = Adverse Childhood Experiences; ADLs = activities of daily living; IADLs = instrumental activities of daily living; MOCA = Montreal Cognitive Assessment. IMIAS = International Mobility in Aging Study; JAGES = Japan Gerontological Evaluation Study; CHARLS = China Health and Retirement Longitudinal Study; NSHAP = National Social Life, Health and Aging Project.

**Table 8:** Studies related to Other Diagnoses or Several Geriatric Syndromes.

Authors	Design, Population	Exposures	Geriatric Syndromes or Other Outcomes	Main Outcome’s Findings	Additional Comments
Wang, 2019 [95], USA	Cross-sectional, 3157 participants aged ≥60, PINE	Child maltreatment (CM) and intimate partner violence (IPV) cases (been physically hurt, insulted, threatened with harm, screamed and cursed at, and a private part touched when it was unwanted) during each age-related period.	Elder abuse (EA) phenotype (psychological, physical, and sexual abuse; financial exploitation; and caregiver neglect) (total 56 items)	CM physical/sexual was associated with increased risks for IPV physical/sexual (OR = 1.86 95% CI 1.02–3.38), EA psychological (OR = 1.70 95% CI 1.20–2.42), and EA financial exploitation (OR = 2.38 95% CI 1.72–3.30).	
Payne, 2020 [96], South Africa	Cross-sectional, 2473 participants aged ≥40, HAALSI	16 Traumatic Experiences (TEs) during their lifetime- five categories: childhood environment, community violence, illness/accident/disaster, social/family environment, and war violence	Depressive symptoms (7-item CES-D) score ≥ 3. PTSD symptoms (Short Screening Scale for PTSD battery; score ≥ 4). Katz ADLs scale. Cognitive impairment (<1.5 SD below the mean in the HAALSI cognitive battery)	TEs were associated with depressive symptoms (OR = 1.08 95% CI 1.03–1.14), PTSD symptoms (OR = 1.17 95% CI 1.07–1.29), ADL limitations OR = 1.11 95% CI 1.03, 1.18).	No association between TEs and later life cognitive impairment
Dorji, 2020 [97], Buthan	Cross-sectional, 337 participants aged ≥60,	WHO ACEs International Questionnaire (13-item): physical or emotional abuse, neglect, someone chronically depressed in a household, incarcerated household member, parental separation or divorce, alcohol or drug abuser or member treated violently in the household, bullying, witnessing of community violence, forced to physical labor, ≥2 weeks had difficulty with basic needs of life	Heart disease, high blood pressure (HBP), diabetes, depression, mobility impairment, lung disease, visual impairment (VI), insomnia, memory decline (MD)	Having ≥ 7 ACEs [vs. 0–2] was associated with higher odds for lung disease (OR = 2.15 95% CI 1.03–4.49), VI (OR = 2.38 95% CI 1.16–4.85), insomnia (OR = 2.35 95% CI 1.11–4.98), MD (OR = 2.30 95% CI 1.10–4.78), HBP (OR = 3.21 95% CI 1.39–7.38), diabetes (OR = 5.12 95% CI 1.06–24.72).	
Lv, 2020 [98], China	Cross-sectional, 6267 participants aged ≥45, CHARLS	Famine fetal exposed, preschool exposed, and school-aged, exposed groups when compared to the non-exposed group	Chronic kidney disease (CKD) was defined as eGFR less than 60 mL/min per 1.73 m^2^.	Fetal exposure to the severe famine was associated with the elevated risk of CKD among male participants (OR = 4.44 95% CI 1.10–17.92)	
Sheffler, 2023 [99], USA	Cross-sectional, 348 participants aged ≥55; North Florida	ACEs, 10-item questionnaire including childhood abuse, parental psychopathology and divorce, and violence in the home occurring before the age of 18.	Sleep quality: Pittsburgh Sleep Quality Index (PSQI). Rating their “sleep quality overall” during the past month from 1 (Very Good) to 4 (Very Bad)	ACEs were associated with significantly worse sleep quality (*B* = 0.156, *p* = 0.008); higher PSQI score indicates lower sleep quality	Adaptive emotion regulation skills had a moderation effect
Ren, 2023 [100], China	Longitudinal; 10,695 participants aged ≥45, CHARLS	ACEs (14- item, self-report) (physical abuse, emotional neglect, family or community violence, parental [divorce, disability, substance abuse, incarceration or death], economic adversity, loneliness, bullying	New-onset pain, falls, chronic diseases, multimorbidity, ADL limitations, and IADL limitations	≥4 ACEs vs. 0 were associated with new-onset pain (HR = 1.57 95% CI 1.37–1.79), falls (HR = 1.84 95% CI 1.55–2.18), chronic diseases (HR = 1.43 95% CI 1.19–1.72), multimorbidity (HR = 1.55 95% CI 1.32–1.83), ADL limitations (HR = 1.85 95% CI 1.54–2.23), and IADL limitations, (HR = 1.46 95% CI 1.24–1.71)	For all outcomes, depressive symptoms was a mediator
Li, 2024 [101], China	Cross-sectional, 12,277 participants aged ≥45, CHARLS	ACEs (12- item) physical abuse, parental mental health, guardians’ bad habits, hunger, feel alone, peer bullied, self-reported health status, the health limitations, death of parents, death of siblings, childhood neighborhood quality, and childhood neighborhood safety	Chronic lung diseases (CLDs) such as chronic bronchitis, emphysema	ACEs that were associated with CLDs include physical abuse (OR = 1.28), parents mental health (OR = 1.50), hunger (OR = 1.20), feel alone (OR = 1.33), peer bullied (OR = 1.56), poor health status (OR = 1.86), health limitation (OR = 1.97), parents’ death (OR = 1.27), siblings’ death (OR = 1.42), unsafe neighborhood (OR = 1.23), and poor neighborhood quality (OR = 1.11)	Depressive symptoms were a partial mediator on the association between CLDs and 7 ACEs
He, 2024 [102], China	Cross-sectional, 24,116 participants aged ≥50, GBCS	ACEs: 5 items reflected separation, traumatic experience, emotional abuse, domestic violence, and parental death	Hemoglobin (Hb) (g/dL). Anemia defined as Hb < 12.0 g/dL for men and <11.0 g/dL for women	Participants with ≥2 ACEs, (vs. 0 ACEs), had lower Hb (β = −0.08, 95% CI −0.12 to −0.03) and higher odds of anemia (OR = 1.26 95% CI 1.01–1.59).	
Cheng, 2025 [103], China	Cross-sectional; 14,582 participants middle-aged, older adults, CHARLS	ACEs (11- item, before age 17)- bullying, corporal punishment, domestic violence, parental substance abuse, poor parental mental health, parental criminality, parental divorce, parental disability, parental death, emotional neglect, and insecurity.	Chronic respiratory diseases [CRD] (COPD and asthma)	Individuals with ≥4 ACEs had OR = 1.48 (95% CI 1.10–1.99) for CRD compared to those with no ACEs	Long sleep duration combined with ACEs increased the risk of CRD
Jin, 2025 [104], China	Cross-sectional; 11,905 participants middle-aged, older adults, CHARLS	ACEs (12- item)- physical abuse, emotional neglect, domestic violence, peer bullying, unsafe neighborhood, parental death, parental disability, sibling death, household mental illness, substance abuse, parental separation or divorce, and incarcerated household members.	Musculoskeletal pain (MSP)	Individuals with ≥4 ACEs had OR = 2.46 (95% CI 2.13–2.83) for MSP compared to those with 0–1 ACEs; depressive symptoms were a partial mediator	Depressive symptoms were a mediator
Chen, 2025 [105], China	Longitudinal; 11,601 participants aged ≥45, CHARLS	ACEs 10 items before the age of 18 (e.g., parental divorce, childhood hunger). Five items for Adverse Adult Experiences (AAEs) (e.g., death of a child, lifetime discrimination)	Dementia diagnosis based on cognitive battery and ADL scale. Stroke (self-reported physician diagnosis)	ACEs (HR = 1.11 95% CI 1.05–1.18) and AAEs (HR = 1.23 95% CI 1.14–1.33) were associated with dementia. AAEs (HR = 1.19 95% CI 1.12–1.26) were associated with stroke.	Depressive symptoms were a mediator
Karhu, 2025 [106], USA	Cross-sectional; 128 patients aged 55 (±16), Cleveland Clinic	ACEs are a 10-item screening tool that assesses exposure to various forms of abuse, neglect, and household dysfunction during the first 18 years of life.	Obstructed Defecation Syndrome (ODS): excessive straining, incomplete rectal evacuation, enemas and laxatives use, vaginal- anal- perineal maneuvers to attempt defecation, and abdominal discomfort/pain caused by obstructed defecation. CCFI measures severity of fecal incontinence.	Patients with ACEs had more severe ODS (not CCFI) scores which correlated with higher HADS anxiety and depression scores.	

Only significant (*p* < 0.05) multivariate results are reported in the table. OR = odds ratios; HR = hazard ratios; β = standardized beta estimated; B = beta estimated; CI = confidence intervals; ACEs = Adverse Childhood Experiences; ADL = activities of daily living; IADL = instrumental activities of daily living; CES-D = Center for Epidemiological Studies–Depression scale; PTSD = Post-Traumatic Stress Disorder. PINE = Population Study of Chinese Elderly in Chicago; HAALSI = Health and Aging in Africa: A Longitudinal Study of an INDEPTH Community in South Africa; CHARLS = China Health and Retirement Longitudinal Study; GBCS = Guangzhou Biobank Cohort Study.

**Table 9:** Summary of Examples for Life course Epidemiology Frameworks * and Main Domains for Exposures.

Life Course Stage	Conceptual Life Course Models/Frameworks	Exposure Domains [References]	Mediators [References]	Geriatric Syndromes
Perinatal Childhood	Critical period	Famine fetal and school-aged exposed [58]		Frailty
Perinatal Childhood	Critical period Social mobility model	Parental illiteracy, economic hardship [42]	Depression Self-rated health	Cognitive impairment
Childhood	Critical period	Adversity [37]	Depression, smoking, low grip strength	Dementia
Childhood	Critical period Social mobility model	Socioeconomic deprivation [80]		Low muscle strength
Childhood	Critical period Pathway models Accumulation of risk Social mobility model	Abuse/neglect Household dysfunction Socioeconomic deprivation Violence [100]	Depressive symptoms	Pain, falls, multimorbidity, functional limitations
Childhood	Critical period Pathway models Accumulation of risk Social mobility model	Abuse/neglect Household dysfunction Socioeconomic deprivation [62]	CRP levels	Depression
Childhood	Critical period	Adversity [57]	Unhealthy lifestyle	Frailty
Childhood	Critical period Pathway models Accumulation of risk	Abuse/neglect [74,77,78,79] Adversity [77,78] Household dysfunction [78,79]	Depressive symptoms [74,77,78,79] Functional decline, multimorbidity, memory difficulty [79]	Falls
Childhood	Critical period Pathway models Accumulation of risk	Abuse/neglect Adversity Household dysfunction [92]	Adult health status and health behaviors	Functional decline
Childhood	Critical period Pathway models Accumulation of risk	Abuse/neglect Adversity Household dysfunction [93]	Chronic conditions	Functional decline
Childhood, young adulthood, late adulthood	Critical period Pathway models Accumulation of risk	Accumulated neighborhood social deprivation [49]		Frailty
Childhood, adulthood	Critical period Pathway models Accumulation of risk	Abuse/neglect Adult violence [91]	Chronic conditions Depression	Functional decline
Childhood, adulthood	Critical period Pathway models Accumulation of risk	Financial adversity, Traumatic events [43]	Depression Self-rated health	Dementia
Childhood, adulthood	Critical period Pathway models Accumulation of risk	Abuse/neglect Household dysfunction Stressful life events [86,87]		Multimorbidity
Childhood, adulthood	Critical period Accumulation of risk Pathway models	Abuse/neglect; Adversity; Loss; Socioeconomic deprivation Household dysfunction Adult violence [63]	Adult socioeconomic deprivation	Depression
Adulthood	Pathway models	Death of a spouse/partner Major financial problems [35]		Dementia
Childhood, adulthood, or older age	Critical period Accumulation of risk	Discrimination at childhood, every day or recent [75,88]		Falls Multimorbidity
Childhood, adulthood, or older age	Pathway models Accumulation of risk	Child maltreatment, intimate partner violence [95]		Intimate partner violence Elder abuse
Older age	Critical period	Discrimination [76]	Depressive symptoms, functional difficulties, multimorbidity	Falls

* Frameworks: accumulation of risk, critical/sensitive periods, pathway models (sequential link between multiple exposures), social mobility model (SES exposures).

## Data Availability

No new data were created or analyzed in this study.
